# Roles of Integrins and Intracellular Molecules in the Migration and Neuritogenesis of Fetal Cortical Neurons: MEK Regulates Only the Neuritogenesis

**DOI:** 10.1155/2013/859257

**Published:** 2013-01-20

**Authors:** Ujjwal K. Rout

**Affiliations:** Departments of Surgery and Physiology and Biophysics, University of Mississippi Medical Center, Room L020, Clinical Service Building, 2500 North State Street, Jackson, MS 39216, USA

## Abstract

The roles of integrin subunits and intracellular molecules in regulating the migration and neuritogenesis of neurons isolated from 16.5 gestation days rat fetal cortices were examined using *in vitro* assays. Results showed that laminin supported the migration of fetal cortical neurons better than fibronectin and that the fetal cortical neurons migrated on laminin using *β*1 and *α*3 integrin subunits which make up the *α*3*β*1 integrin receptor. On fibronectin, the migration was mediated by *β*1 integrin subunit. Perturbation of src kinase, phospholipase C, or protein kinase C activity, inhibition of IP3 receptor mediated calcium release, or chelation of intracellular calcium inhibited both migration and neuritogenesis, whereas inhibition of growth factor signaling via MEK inhibited only the neuritogenesis. The detection of *α*1 and *α*9 transcripts suggested that the migration of fetal cortical neurons may also be mediated by *α*1*β*1 and *α*9*β*1 integrin receptors. Results showed that calcium may regulate migration and neuritogenesis by maintaining optimum levels of microtubules in the fetal cortical neurons. It is concluded that the fetal cortical neurons are fully equipped with the integrin signaling cascade required for their migration and neuritogenesis, whereas crosstalk between the integrin and growth-factor signaling regulate only the neuritogenesis.

## 1. Introduction

During brain development, postmitotic neurons migrate from their site of origin to distant places, differentiate, and make connections forming different layers of the cortex. Defects in this process results in abnormal neuronal positioning and connections in the brain, which may cause neurobehavioral problems later in life [1]. Neuritogenesis, an early step of neuron differentiation, is the synthesis of multiple growth cone tipped extensions (neurites) that ultimately form the axons and dendrites of neurons [2]. Mechanisms that regulate the migration and differentiation of neurons are not fully understood. 

Cell surface integrin receptors, each consisting of an *α* and a *β* subunit, play critical roles in the glial-guided migration of neurons in the brain [3]. The extracellular domains of the receptor subunits bind with extracellular matrix (ECM) proteins (such as fibronectin and laminin) and the cytosolic domains of *β* subunits interact with kinases, adaptor molecules, and the cytoskeleton [4]. These interactions facilitate the “outside-in” and the “inside-out” signaling across the cell membrane by the integrin heterodimers [5] that may lead to cell migration and neuritogenesis [6].

Integrin receptors with explicit combinations of *α* and *β* subunits interact with specific ECM proteins [7]. Developing brains express *β*1, *α*1, *α*3, *α*4, *α*5, *α*6, and *αv* integrin subunits which may form *α*1*β*1, *α*3*β*1, *α*4*β*1, *α*5*β*1, *α*6*β*1, and *αvβ*1 integrin receptors [8, 9]. Of all these receptors, the *α*1*β*1, *α*3*β*1, and *α*6*β*1 receptors interact with laminin, whereas *α*4*β*1, *α*5*β*1, and *αvβ*1 receptors interact with fibronectin [7].

Manipulation of integrin subunits *α*3 and *α*5 genes in mice causes abnormal positioning of neurons in the cerebral cortex, and nullifying the *α*6 gene results in cortical laminar defects [3, 10–12]. These studies suggest that integrin receptors consisting of *β*1 subunit (*α*3*β*1, *α*5*β*1, and *α*6*β*1) are involved in the migration of neurons in the brain. This is also in accordance to a large number of reports describing the role of integrin receptors consisting of *β*1 subunit in neuron migration and neuritogenesis [13–16]. However, studies involving the ablation of *β*1 integrin subunit gene in mice [17–20] suggest that this integrin subunit is required for the glial end feet anchorage and is not essential for neuron-glia interactions and glial-guided migration of neurons during brain development. Moreover, additional studies indicate that *β*1 integrin subunit is involved not in the migration but the differentiation of glia and neurons [2, 20–22]. Therefore, to date, the studies on the role of *β*1 subunit containing integrins (*α*3*β*1, *α*5*β*1, and *α*6*β*1) in the migration of fetal brain neurons have been largely inconclusive. Experiments with antibodies against integrin subunits or inhibitors of integrin signaling in slice cultures [23–26] are also not free from the indirect effects that may influence the migration and differentiation of neurons. 

Therefore, in the present study, neurons were isolated from 16.5 gestation days fetal cerebral cortices of rats for the direct examination of the role of molecules that are known to regulate the integrin-mediated cell migration. Because different ECM molecules are secreted by radial glial cells during development [27, 28], initially different concentrations of laminin and fibronectin were examined on the migration of fetal cortical neurons. The propensity of *α* subunits forming integrin receptor/s with the *β*1 subunit to support migration was examined by using the same concentration of antibodies against different *α* subunits at which the migration of neurons was significantly inhibited by antibody against *β*1 subunit. Roles of different intracellular molecules (src kinase, phospholipase C, calcium, protein kinase C, MEK kinase, and microtubules) that are known to support integrin-mediated migration of different cell types [29–31] were examined on neuron migration by using pharmacological inhibitors or intracellular calcium chelator. The roles of *β*1 and *α*3 integrin subunits, the intracellular molecules, and the calcium chelator were also examined on neuritogenesis. 

## 2. Materials and Methods

### 2.1. Animals and Compliance

Time pregnant Long-Evans rats were obtained from Charles River (Boston, MA). Animals were housed in the animal facility of the University of Mississippi Medical Center on chow and water *ad libitum *and used for studies according to a protocol approved by the Institutional Animal Care and Use Committee of the University of Mississippi Medical Center. 

### 2.2. Reagents

Hanks' balanced salt solution (HBSS), Dulbecco's modified eagles medium and F12 medium (DF), B-27, Trypsin inhibitor, mouse laminin, bovine fibronectin, nuclear stain Hoechst 33342, magnesium sulphate, Trypsin, and cell culture tested Dimethyl sulfoxide were purchased from Sigma-Aldrich Chemical Company, St. Louis, MO. Molecular weight markers used in western blot studies were obtained from Bio-Rad Laboratories (Hercules, CA). 

Monoclonal antibodies against the extracellular domains of integrin subunits were obtained from different commercial sources in no-azide formulations or freed from sodium-azide using Nanosep centrifugal devices (PALL Life Sciences, Ann Arbor, MI). Additional information on these antibodies as well as those used in the immunofluorescent staining and western blotting experiments (see below) is provided in Table 1. Inhibitors against different enzymes that are known to regulate integrin signaling in different cell types and modulators of intracellular calcium levels are mentioned in Table 2. 

### 2.3. Preparation of Dissociated Neurons

 Dissociated neurons were prepared from cortices as described previously [39] with some modifications. Briefly, on gestation days 16.5, pregnant rats were deeply anesthetized with isoflurane, fetuses were removed from uteri, and cerebral cortices of fetal brains were dissected out as described previously [40]. Cerebral cortices were incubated in HBSS containing 0.1% Trypsin for 15 min at 37°C. Cortices were triturated in HBSS containing 0.025% DNase I, 0.2% Trypsin inhibitor, 0.2% BSA, and 12 mM MgSO_4_ and centrifuged at 160 g. The resulting pellet was suspended in HBSS and centrifuged. Supernatant was discarded and the pellet was suspended in the culture medium DMEM/F-12 (1 : 1) containing 0.2% B-27 to estimate the number using a hemacytometer (Hausser Scientific, Horsham, PA). The neurons were used for migration assays and also cultured on laminin-coated wells of 8 chambered glass slides (Thermo Fisher Scientific, Houston, TX) for immunofluorescent microscopy and neuritogenesis assays or in flasks (Fisher Scientific) for western blotting and RNA isolation. 

### 2.4. *In Vitro* Neuron Migration Assay

The Boyden Chamber assay that has been successfully used for estimating the migration of fetal brain neurons previously by different laboratories [39, 41–43] was used for this study. Specifically, the Boyden chamber assay for studying fetal cerebral cortical neurons of Maeda and Noda (1998) [39] was used with some modifications. Transwell inserts (diameter 6.5 mm), each consisting of polycarbonate membranes with 3.0 *μ*m pore size with pore density 2 × 10^6^/cm^2^ (Fisher Scientific; Pittsburgh, PA) were used for migration assays. Undersurface of membranes of Transwell inserts was first coated with Poly D Lysine (10 *μ*g/mL) overnight and then with solution (100 *μ*L) of laminin, fibronectin, or only water (diluent of laminin or fibronectin solutions) and dried overnight in sterile conditions prior to use. In all migration assays, the lower chamber contained 500 *μ*L of culture medium (controls) or medium containing antibody against integrin subunit, inhibitor, or calcium chelator (see below) and the upper chamber contained 200 *μ*L of medium containing 150,000 dissociated neurons. Plates containing the Boyden chamber assemblies were incubated for 18 h in an incubator (37°C, 5% CO_2_, humidity 99%). At the end of incubation, membranes were treated with chilled 4% paraformaldehyde solution for 20 min and the neurons on the upper surface of the membranes were removed by rubbing with cotton swabs twice. Preliminary experiments were conducted to ensure that rubbing technique removes all neurons from the upper surface of membrane and the incubation time that provided adequate number of neurons for migration assays. Membranes were cut and processed for the immunofluorescent detection of neuronal nuclei and neuronal marker MAP2 expression as described below (see Section  2.10). All migration assays were performed at least three times with two Boyden chamber assemblies per experimental conditions. Images of different fields vertically and across (total 9) of each membrane were captured at 10x magnification. Total numbers of nuclei from each image of controls and experimental groups were counted using the counting tool of the Metamorph software to determine the number of nuclei per membrane for analysis.

### 2.5. ECM Effects on the Migration of Neurons

Relative roles of ECM proteins (laminin and fibronectin) on the migration of dissociated neurons were examined by coating Poly-D lysine treated membranes with different concentrations of laminin (1, 5, 10, 20, and 50 *μ*g/mL) or fibronectin (10, 20, 50, 100, and 200 *μ*g/mL) in sterile water. Medium containing neurons (150,000) were added in the upper chamber and the lower chambers contained medium only. Chambers were incubated for 18 h and the neurons on the undersurface of the membranes were counted as described above.

### 2.6. Antibody Effects on the Migration of Neurons

Relative roles of different integrin *α* subunits with respect to *β*1 subunit on the migration of neurons were examined using functional blocking antibodies. Azide-free monoclonal antibody against different integrin subunits, control IgG, or IgM were added in the medium of lower chamber. Control chambers contained only medium in both upper and lower chambers. Upper chamber contained neurons (150,000) in the medium (200 *μ*L). Neurons on the lower surface of membranes were counted at the end of 18 h incubations as described above. Initial experiments were conducted with monoclonal antibodies against *β*1 integrin subunit to determine the concentration (50 nMole) that significantly inhibited the migration of neurons at *P* < 0.05 on laminin (10 *μ*g/mL). Relative effects of monoclonal antibodies against additional integrin subunits at (50 nMole) were tested on the migration of neurons on laminin (10 *μ*g/mL) or fibronectin (100 *μ*g/mL) coated membranes. These antibodies (see Table 1) are tested for their efficacy by the supplier and also reported for their interaction with integrin subunits on cell surface and inhibition of cell adhesion [44–50]. 

### 2.7. Effects of Inhibitors or Calcium Chelator on the Migration of Neurons

Involvements of different intracellular signaling molecules in the migration of neurons were examined using pharmacological inhibitors. This included inhibitors PP2, U-73122 (U2), PD98059 (PD), Calphostin C, 2-APB, and Ruthenium Red against Src family of tyrosine kinases, PLC activation, MAP kinase kinase, Protein kinase C, IP3-induced calcium release, and calcium induced calcium release from Ryanodine receptors, respectively. Role of intracellular calcium on migration was examined using the chelator BAPTA-AM. Inhibitors (PP2, U2, 2-APB, and PD) or control compounds U-73343 (U3) and PP3 and intracellular calcium regulators (BAPTA-AM and Ruthenium Red) were added in the medium of lower chamber. Upper chamber contained 150,000 neurons suspended in the medium only. The concentrations of these pharmacological compounds, their target and relevant references, are mentioned in Table 2. 

### 2.8. Effects of Antibodies, Inhibitors, or Calcium Chelator on Neuritogenesis

Roles of integrin subunits, intracellular signaling molecules and calcium on neuritogenesis were examined using antibodies, pharmacological inhibitors, and calcium chelators. Wells of 8 chambered glass slides were coated with gelatin (12 h at 4°C) followed by laminin (10 *μ*g/mL) (12 h at 4°C). Dissociated neurons (100,000) were cultured in wells in the absence (control) or presence of monoclonal antibody (Table 1), inhibitor, or calcium modulators (Table 2) for 20 h and bright-field images (6/well = 3 vertical and 3 across) of neurons were captured. High contrast images were captured for visualization and tracing of neurites with the tools of Metamorph software. Experiments were repeated to collect data from at least 3 wells per control and treatment conditions (18 images/treatment condition). 

### 2.9. Effects of BAPTA on the Expression of Microtubules

Role of intracellular calcium on the expression of microtubules was examined using different concentrations of calcium chelator BAPTA-AM (0, 5, 10, and 20 *μ*M) by immunofluorescent microscopy (see below).

### 2.10. Immunofluorescent Microscopy

Expression of neuron markers (NeuN and MAP2) and microtubules were examined in the dissociated neurons by immunofluorescent microscopy. Briefly, at the completion of paraformaldehyde treatments, neurons on the undersurface of membranes or in the wells of eight chambered slides were treated with Triton (0.1%) for 10 min and rinsed with PBS prior to incubating with the primary antibody solution in Phosphate Buffer Saline (PBS) containing Bovine Serum Albumin (BSA) (1 mg/mL) overnight at 4°C. Dilution of primary antibodies for the detection of NeuN, Microtubules, and MAP2 in PBS-BSA solutions were 1 : 10, 1 : 200, and 1 : 1000, respectively. Membranes or wells were rinsed with PBS three times (15 min each) and then incubated with Alexa dye conjugated secondary antibody (10 *μ*g/mL) and nuclear stain Hoechst 33342 (dilution 1 : 200). Fluorescent images were captured using a Nikon Eclipse (TE-2000 U) Microscope. 

### 2.11. Reverse Transcription and Polymerase Chain Reactions (RT-PCR)

Expression of transcripts representing different integrin subunits in the fetal cortical neurons, cultured on laminin-coated flasks for 7 h, was examined using RT-PCR in three occasions. In each experiment, the total RNA (1 *μ*g) isolated from the neurons using Trizol-reagent (Invitrogen, Carlsbad, CA) was subjected to complementary DNA (cDNA) synthesis using a Reverse transcription III kit (Invitrogen). Equal amounts (50 ng equivalent) of cDNA samples were amplified by PCR. The primer sequences (Table 3) were either obtained from the literature [51–53] or subjecting the rat or homologous regions of mice and human cDNA sequences (GenBank) to Primer3 software (http://flypush.imgen.bcm.tmc.edu/primer/primer3_www.cgi/). Authenticity of these primers was tested by PCR using cDNA from rat and human cell lines (rat PC12 and cardiac myoblasts; human trophoblast and colon cancer cell lines) that produced expected size amplicons with no spurious bands. Identity of amplicons representing *α*3 and *α*9 integrin subunit was confirmed by commercial sequencing (Retrogen, Inc., SanDiego, CA).

### 2.12. Western Blotting

A previously described western blotting methodology [40] was used to examine the expressions of phosphorylated PLC-*γ*1 in the dissociated neuronal preparations cultured on laminin-coated flasks for 6 h. Clear lysatefrom neurons containing 10 *μ*g proteins were denatured by boiling in the presence of lane marker buffer (Pierce) and subjected to electrophoresis on a 7.5% Polyacrylamide gel containing sodium dodecyl sulphate. Molecular weight markers (Bio-Rad) were also loaded in adjacent lanes. At the end of electrophoresis separated proteins were transferred on to nitrocellulose membranes and probed with primary antibodies against phosphorylated PLC-*γ*1 and *β*-Actin (Table 1). Membranes were washed and exposed with Peroxidase conjugated secondary antibodies. Bands were detected using Amersham Hyper film ECL (GE Healthcare Ltd., Buckinghamshire, UK). The experiment for the detection of phosphorylated PLC-*γ* was tested using 3 different neuron preparations. 

### 2.13. Statistical Analysis

Analysis of the neuronal migrations and neuritogenesis was conducted by ANOVA using SPSS software (SPSS, Chicago, IL). Neuron migrations were assayed by comparing the mean ± standard errors of mean values of the number of neuronal nuclei from 9 fields under each Boyden membrane of total 6 to 9 membranes per treatment group. The neuritogenesis was examined by comparing the mean ± standard errors of mean values of the neurite lengths from 6 fields per well (total 3 wells of each control and treated groups). Differences in Mean ± Standard errors of mean values at *P* < 0.05 were considered significant. 

## 3. Results

### 3.1. Isolation of Fetal Cortical Neurons

**Figure 1:**
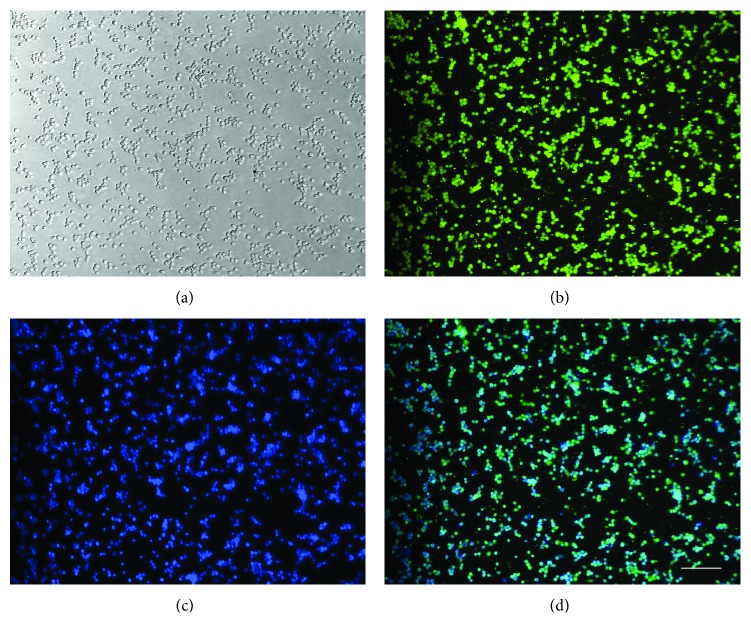
Dissociated neurons in culture. Dissociated neurons at 2 h of plating on laminin-coated plates (a) immunostained for the neuronal marker NeuN (b) and nuclear stain Hoechst (c). Image (d) shows the overlaps of images (b) and (c). Bar100 microns. Note, Hoechst stained nuclei are out of focus because all images are captured at the same plane as (a).

**Figure 2:**
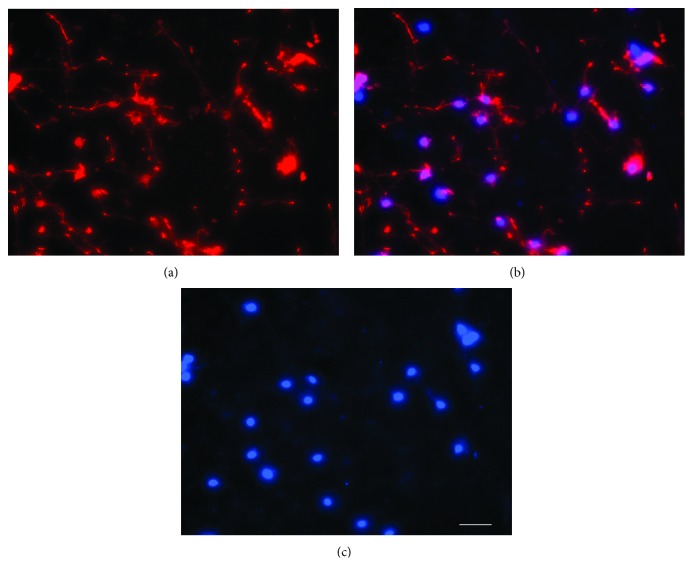
Neurons at the undersurface of membrane. Neurons migrated at the undersurface of membrane expressed neuronal marker MAP2 (a). Neuronal nuclei stained with Hoechst (c). Overlapping images (a) and (c) are shown in (b). Bar 100 microns.

The number of neurons isolated per pair of cortices by the described method was approximately 5 × 10^7^ neurons (*N* = 7). These neurons attached with the laminin-coated wells and majority (approximately 95%) expressed neuronal marker NeuN (Figure 1), and when incubated in Boyden chambers for 18 h, migrated at the undersurface of the membranes and expressed neuronal marker MAP2 (Figure 2).

### 3.2. ECM Effects on the Migration of Neurons (Figure 3)

Migration of cortical neurons was higher on membranes coated with 5, 10, 20, or 50 *μ*g/mL laminin than those coated with only 1 *μ*g/mL laminin or no laminin. Highest migration of neurons occurred on membranes coated with 10 *μ*g/mL laminin. The migration of neurons on membranes coated with 20, 50, 100, or 200 *μ*g/mL fibronectin was higher than those coated with 10 *μ*g/mL fibronectin or no fibronectin. The migration of neurons on membrane coated with 100 or 200 *μ*g/mL fibronectin was not significantly different (*P* > 0.05). Further studies on the migration of neurons were conducted with membranes coated with Poly-D lysine followed by 10 *μ*g/mL laminin or 100 *μ*g/mL fibronectin (see below). 

### 3.3. Antibody Effects on the Migration of Neurons (Figure 4)

Antibody against *β*1 or *α*3 integrin subunit significantly decreased migration of neurons on laminin-coated membranes (*P* < 0.05). Antibody against *α*6 subunit did not alter the migration of neurons on laminin-coated membranes. Moreover, the migration of neurons was not altered by control antibody (IgG or IgM) or the antibody against the *αv* subunit (negative control on laminin) on laminin-coated membranes. On fibronectin-coated membranes, only antibody against *β*1 subunit inhibited the migration (*P* < 0.05). The migration of neurons on fibronectin-coated membranes was not altered by control antibodies (IgG or IgM), and antibodies against *β*3 (negative control since not expressed), *α*4, *α*5, or *αv* subunits. 

### 3.4. Effects of Inhibitors and Calcium Modulators on the Migration of Neurons (Figure 5)

PP2 (the inhibitors of Src kinase activation), U2 (the inhibitor of PLC*γ* activation), and the light-activated Calphostin (the inhibitor of PKC activation) inhibited the migration of neurons on both laminin- and fibronectin-coated membranes (*P* < 0.05). Migrations of neurons were not significantly altered in the presence of MAP Kinase Kinase (MAPK/ERK kinase or MEK) inhibitor PD, control compounds U3 or PP3 on laminin, or fibronectin-coated membranes (*P* > 0.05). The inhibitor of IP3 receptor-induced calcium release from intracellular stores (2-APB) and the intracellular calcium chelator BAPTA-AM inhibited the migration both on laminin- and fibronectin-coated membranes. Ruthenium Red, the inhibitor of intracellular calcium-induced calcium release from Ryanodine receptor containing calcium stores, did not alter the migration of neurons.

### 3.5. Effects of Antibodies, Inhibitors, and Calcium Modulators on Neuritogenesis

Antibodies against *β*1 or *α*3 subunit inhibited neuritogenesis of fetal cortical neurons. Neuritogenesis was not inhibited in the presence of control antibodies (IgG) at 50 nM (Figure 6(a)) and even at higher concentration (100 nM), (Figure 6(b)). BAPTA-AM completely abolished the neurite formation at higher concentration (10 *μ*M), (Figure 6(a)). The inhibitors of Src kinase (PP2), PLC-*γ* respectively (U2), IP-3 induced calcium release (2-APB), Protein kinase C (activated calphostin), MAP kinase kinase (PD), and the calcium chelator (BAPTA-AM) significantly reduced the neurite lengths (Figure 6(c)). Neuritogenesis was not inhibited in the presence of control compounds of pharmacological inhibitors (PP3 and U3) or the inhibitor of Ryanodine receptor mediated release of intracellular calcium (Ruthenium Red) (Figure 6(c)). BAPTA-AM decreased the expression of microtubules in cortical neurons (Figure 7).

### 3.6. Expression of Phosphorylated PLC*γ*1 and Integrin Subunit mRNA Species in Dissociated Neurons

Western blotting experiments with lysate prepared from neurons at 6 h of culture provided evidence for the presence of PLC-*γ*1 in its phosphorylated state at tyrosine 783 (Figure 8). Neurons at 6 h of culture expressed mRNA for *β*1, *α*1, *α*3, *α*4, *α*5, *αv*, and *α*9 integrin subunits (Figure 9). 

## 4. Discussion

Cortical neurons isolated from rat fetal (GD16.5) brains expressed subunits for integrin receptors and responded to cues of both laminin and fibronectin during their migration *in vitro*. These neurons utilized the intracellular signaling molecules and cytoskeletal elements that are known to transmit integrin receptor signaling during cell migration and differentiation. At the same concentrations, the effects of inhibitors and intracellular calcium modulators were more robust on neuritogenesis than on migration. Results showed that growth factor signaling via MEK is only required for neuritogenesis. The connotations of these findings are discussed below. 

**Figure 3:**
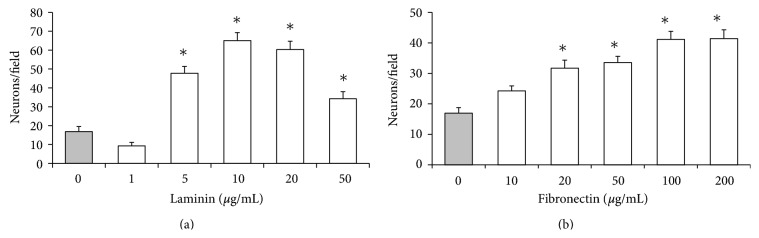
ECM effects on the migration of neurons. Neurons per field of Boyden membrane coated with laminin (a) or fibronectin (b). ∗Migration of cortical neurons were significantly (*P* < 0.05) higher on membranes coated with laminin at 5, 10, 20, and 50 *μ*M or 20 to 200 *μ*M fibronectin compared to those coated with only Poly-D lysine (shaded bar). Migration of neurons was highest at 10 *μ*M laminin but it was not significantly different than those at 20 *μ*M laminin. Migration of neurons on fibronectin-coated membranes increased with concentration to reach its maximum at 100 *μ*M that was not significantly (*P* > 0.05) different than membranes coated with 200 *μ*M fibronectin.

Membranes coated with laminin or fibronectin enhanced the migration of fetal cortical neurons (Figure 3). It was evident that laminin at a 10-fold lower concentration supported the migration better than fibronectin suggesting that laminin may be a better substrate for the migration of fetal brain neurons. This observation is consistent with the migration pattern of neuronal precursors derived from embryonic stem cells [14]. Optimum adhesion with the ECM is critical for the normal migration of cells [54, 55]. Therefore, the enhanced migration of neurons with the increased concentration of laminin and its downregulation with further increase in laminin (Figure 3(a)) indicates that the pace and the direction of neuron migration in the fetal brain may be regulated by the amount of specific ECM molecule secreted by glial cells on the migratory route. 

**Figure 4:**
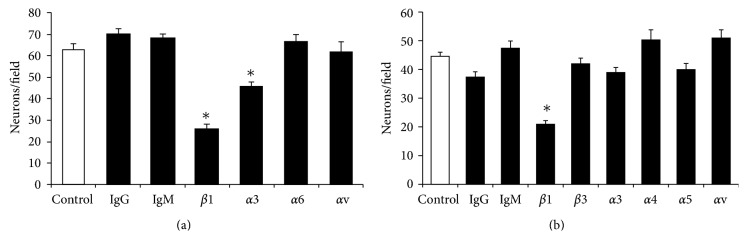
Effects of antibodies on the migration of fetal cortical neurons. Monoclonal antibodies (shown below) against *β*1 and *α*3 integrin subunits significantly (^*^ *P* < 0.05) inhibited the migration of neurons (Neurons/field) on membranes coated with laminin (10 *μ*g/mL) (a). The migrations of neurons on laminin-coated membranes were not significantly (*P* > 0.05) altered by antibody against *α*6 or *αv* subunit (the negative control) and control antibodies (IgG or IgM). The migrations of neurons on fibronectin (100 *μ*g/mL) coated membranes were significantly (^*^ *P* < 0.05) inhibited by the antibody against *β*1 integrin subunit only (b). The migrations of neurons on fibronectin-coated membranes were not altered by control antibodies (IgG or IgM) at *P* > 0.05.

Inhibition studies with monoclonal antibodies to examine the relative roles of different integrin subunits on migration suggested that the receptor consisting of *α*3 and *β*1 subunits (*α*3*β*1) primarily regulates the migration of fetal cortical neurons on laminin (Figure 4(a)). This is also in accordance with previous observations [3, 10, 15]. In addition, the lack of effects of antibody against the *α*6 integrin subunit on the *α*6*β*1 mediated migration is also consistent with the absence *α*6 subunit in fetal cortical neurons (Figure 9) and the lack of any direct evidence for its role in the migration of fetal cortical neurons [11]. On fibronectin-coated membranes, only the antibody against *β*1 subunit inhibited the migration of neurons and antibodies against the *α* subunits (*α*3, *α*4, *α*5, or *αv*) that are the constituent of fibronectin-binding receptors (*α*3*β*1, *α*4*β*1, *α*5*β*1, and *αvβ*1) did not inhibit the migration (Figure 4(b)) although the RT-PCR (Figure 9) results confirmed their expression. This finding indicates that the migration of fetal cortical neurons on fibronectin may be regulated by *β*1 subunit containing integrin receptor/s other than *α*3*β*1, *α*4*β*1, *α*5*β*1, and *αvβ*1. It may include the fibronectin binding receptor *α*8*β*1 that also interacts with ECM tenascin. The role of tenascin in the glial guided migration of fetal cortical neurons is further supported by the fact that tenascin is expressed by the radial glia in the developing brain [56] and transcripts of *α*9 subunits are expressed in fetal cortical neurons (Figure 9), which also forms the tenascin interacting *α*9*β*1 integrin receptor. 

While the antibody against *αv* subunit is reported to inhibit the migration of fetal cortical neurons in an imprint assay [3], it failed to inhibit the migration on fibronectin-coated membranes in the Boyden assay (Figure 4(b)). The role of integrin receptors consisting of *αv* subunits (*αvβ*1, *αvβ*5, or *αvβ*8) in the migration of fetal cortical neurons is further lessened by the fact that vitronectin, the substrate for these receptors, is primarily expressed in the proximity of blood capillaries [57] and the ablation of *αv* subunit expression by gene manipulation only disturbs the development of blood vessels and axonal survival but not neuronal positioning [58]. It is surprising, however, to find no effect of antibody against *α*5 subunit on migration of neurons (Figure 4(b)) because its genetic manipulation at GD15.5 in mice causes laminar defects [12]. The exact reason for this discrepancy is yet to be known. 

Application of different pharmacological inhibitors revealed that the intracellular molecules that mediate integrin signaling (Figure 10) and known to regulate migration of various cell types [30] are fully functional in the fetal cortical neurons. These included Src Kinase, that is activated by focal adhesion kinase following the interaction of integrin receptors with the ECM [59]; Phospholipase C*γ*, that is activated by the activated Src Kinase, and hydrolyses membrane protein PIP2 (phosphotidal inositol biphosphate) into Inositol (1,4,5)-triphosphate (IP3) and Diacylglycerol (DAG) [60, 61]; Protein Kinase C, that is activated by the DAG [62], and the calcium released from the IP3 receptor containing stores [63]. 

The PLC-*γ*1 interacts directly with the cytoplasmic tail of the *β*1 integrin subunit and induces cytoskeletal organization and integrin mediated migration of cells [61]. Integrin mediated cell adhesion promotes rapid autophosphorylation [64] of Tyr-397 residue in focal adhesion kinase (FAK) promoting its interaction with the C-terminal SH2 domain of PLC-*γ*1 and associating with the Src kinases at the site of integrin-ECM interactions [64]. This causes phosphorylation of Tyrosine residue 783 (Tyr783) and the activation of PLC-*γ*1 required for the integrin mediated upregulation of intracellular calcium and migration of cells [65]. Western blotting data (Figure 8) confirmed the presence of the activated form of PLC-*γ*1 isoform in the fetal cortical neurons suggesting its possible interaction with the cytoplasmic tail of the *β*1 integrin subunit in fetal cortical neurons. 

**Figure 5:**
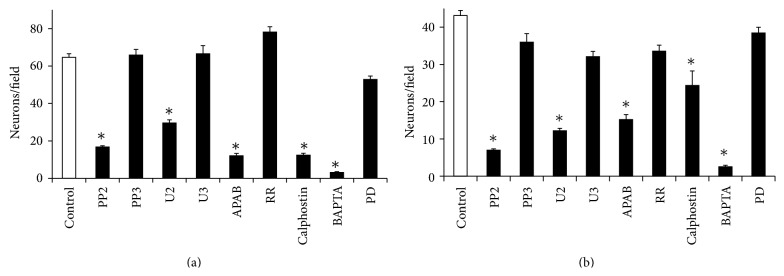
Effects of inhibitors on the migration of cortical neurons. Inhibitors (shown below) of Src kinase (PP2) and Phospholipase C*γ* activity (U2) inhibited the migration of fetal cortical neurons (Neurons/field) on laminin-(a) or fibronectin-(b) coated membranes (^*^ *P* < 0.05). No significant changes in the migration of neurons occurred in the presence of control compound PP3 or U3 (*P* > 0.05). Inhibitor of protein kinase C (light-activated Calphostin), inhibitor of IP3 mediated calcium release (2-APB), and intracellular calcium chelator (BAPTA-AM) inhibited the migration of neurons significantly (^*^ *P* < 0.05) on laminin-(a) or fibronectin-(b) coated membranes. Ruthenium Red (inhibitor calcium-induced calcium release) and PD (inhibitor of Mitogen activated kinase kinase) did not alter the migration of neurons on laminin-(a) or fibronectin-(b) coated membranes (*P* > 0.05).

Previous studies report that PKC*δ* and atypical PKC regulate migration of neuronal precursors in the fetal cerebral cortex [66, 67] and different PKC isoforms (PKC*α*, PKC*β*I, PKC*β*II, PKC*δ*, PKC*ε*, and PKC*θ*) support migration of cells by associating with *β*1 integrin subunit [68]. Robust reduction of migration in the presence of PKC inhibitor (Calphostin C) (Figure 5) shows that the activated state of PKC isoform/s in fetal cortical neurons is required for their migration on both laminin and fibronectin. 

Even though the integrin and growth factor receptor cross-talk is reported to play a significant role in the migration of various cell types [31], the MEK inhibitor PD98059 did not alter the migration of fetal cortical neurons significantly (Figure 5) but rigorously reduced neuritogenesis (approximately 14 fold) (Figure 7(c)). This suggests that the migration of fetal cortical neurons is primarily regulated by integrin mediated pathways, whereas the differentiation of these neurons is regulated by both integrin and growth factor signaling (Figure 10). The sustained expression of certain adaptor/s (such as ILK, PINCH, and Paxillin) that are required for cross-talk between integrin and growth factor receptors during differentiation but not migration of fetal cortical neurons may instruct such effects [69]. Interestingly, ILK is required for the differentiation of Bergmann glia during the cerebellar development [22]. 

The reduced migration of neurons in the presence of BAPTA-AM (Figure 5) confirmed previous observations that intracellular calcium is an indispensable mediator for the migration of fetal brain neurons [63]. On the other hand, no effects of high concentration (50 *μ*M) of Ruthenium Red on migration are in contrast to the finding that integrin-mediated signaling mobilizes calcium from the Ryanodine Receptor gated stores [70].

**Figure 6:**
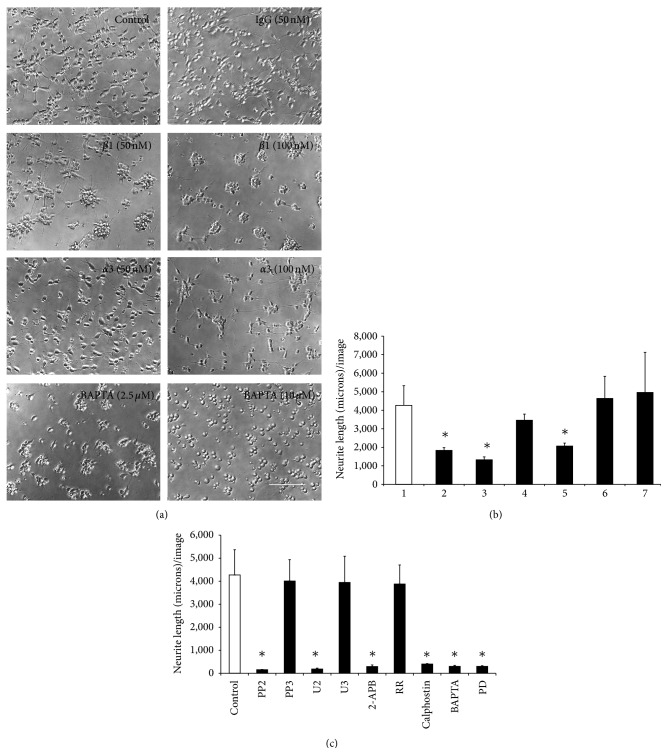
Effects of antibodies, calcium modulators, and pharmacological inhibitors on neuritogenesis. (a) Representative images showing neuritogenesis in the absence (control) or presence of control IgG (50 nMole), antibodies against *β*1 (50 or 100 nMole) or *α*3 (50 or 100 nMole) integrin subunits on laminin. BAPTA-AM at low concentration (2.5 *μ*M) inhibited neuritogenesis and at higher concentration (10 *μ*M) totally abolished neuritogenesis. Bar150 microns. (b) Mean + standard error of mean values of neurite lengths/image in untreated neurons (1) and those treated with control IgG, monoclonal antibody against *β*1 or *α*3 integrin subunit at 50 nMole or 100 nMole concentration. Neurite lengths reduced significantly (^*^ *P* < 0.05) in neurons treated with antibody against *β*1 integrin subunit at both 50 nMole (2) and 100 nMole (3) concentrations compared to control (1). Neurons treated with monoclonal antibody against *α*3 integrin subunit at 50 nMole (4) were lower than the control (1) but was not statistically significant (*P* > 0.05). Neurons treated with monoclonal antibody against *α*3 integrin subunit at 100 nMole (5) significantly inhibited the neuritogenesis (^*^ *P* < 0.055). Neuritogenesis was not altered in presence of 50 (6) or 100 nMole (7) control IgG (*P* > 0.05). (c) Mean + standard error of mean values of neurite lengths/image of neurons treated with PP2, PP3, U2, U3, 2-APB, activated Calphostin, BAPTA-AM (2.5 *μ*M), and PD for 22 h in culture (filled bars) were significantly different (^*^ *P* < 0.05) from untreated neurons (blank bar) and negative controls (PP3 or U3) (filled bars). No significant changes in neurite lengths (*P* > 0.05) per image were recorded in neurons treated with RR.

**Figure 7:**
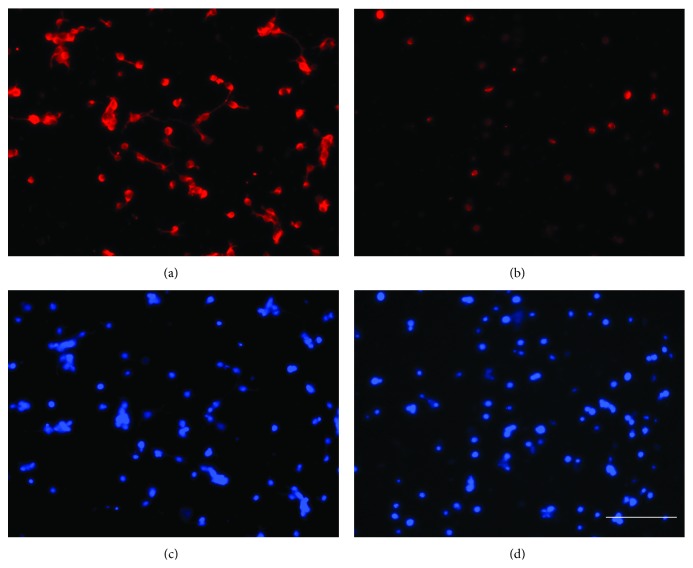
BAPTA-AM reduced microtubule expression. Microtubule expression in the untreated neurons (a) and those treated with BAPTA-AM (10 *μ*M) (b) in culture. Panel (c) and (d) shows nuclear stain in the control and BAPTA-AM treated neurons. Bar 100 microns. Note, Hoechst stained nuclei in (c) and (d) are out of focus because images are captured at the same plane as (a) or (b), respectively. Bar 100 microns.

**Figure 8:**
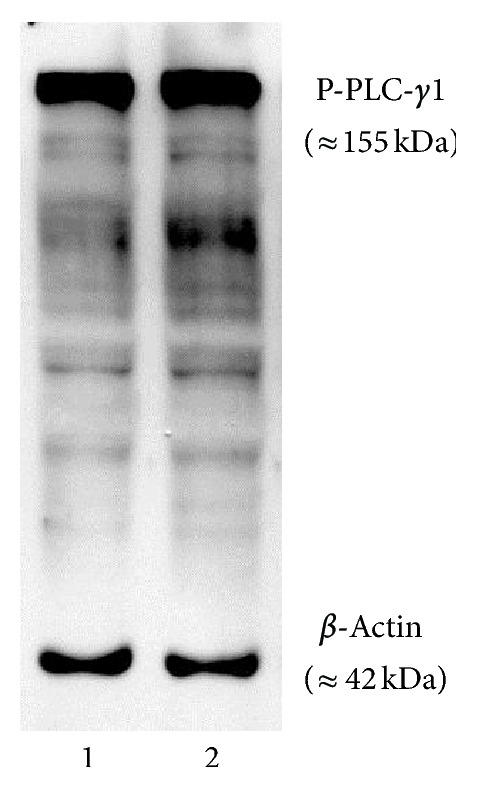
Western blotting detection of phosphorylated PLC-*γ*1 in neurons. Phosphorylated form of PLC-*γ*1 protein at tyrosine 783 was detected in the neurons at 6 h of culture on laminin simultaneously with *β*-actin. Lanes 1 and 2 represent lysate loaded from different neuronal preparations. Molecular weight sizes of phosphorylated form of PLC-*γ* isoform and *β*-actin are shown in parentheses.

**Figure 9:**
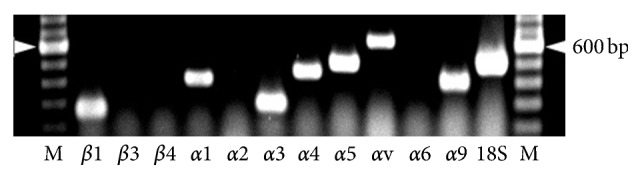
Transcripts of integrin subunits in the fetal cortical neurons. Ethidium bromide stained PCR products after agarose gel electrophoresis. Target mRNA species are shown at the bottom of the image. The 600 bp band of the 100 bp marker (M) is shown by arrows heads on sides.

The neuritogenesis data showed that the integrin receptor consisting of *α*3 and *β*1 subunits also regulates neuritogenesis in rat fetal cortical neurons. This is in accordance to previous reports [2, 14]. The present study further shows that the signaling molecules that mediate the migration of fetal cortical neurons (Src kinase, PLC-*γ*, Protein kinase C, IP-3 induced calcium release, and the intracellular calcium itself) also regulate the process of neuritogenesis albeit more robustly than migration (Figures 5 and 6(c)). Furthermore, inhibition of neuritogenesis by BAPTA at a moderate concentration (2.5 *μ*M), its total abolition at higher concentration (10 *μ*M) (Figure 6(a)), and reduction in microtubule expression (Figure 7) suggest that intracellular calcium not only regulates the dynamics of microtubule dynamics [71] but may also maintain its intracellular levels.

## 5. Conclusions 

**Figure 10:**
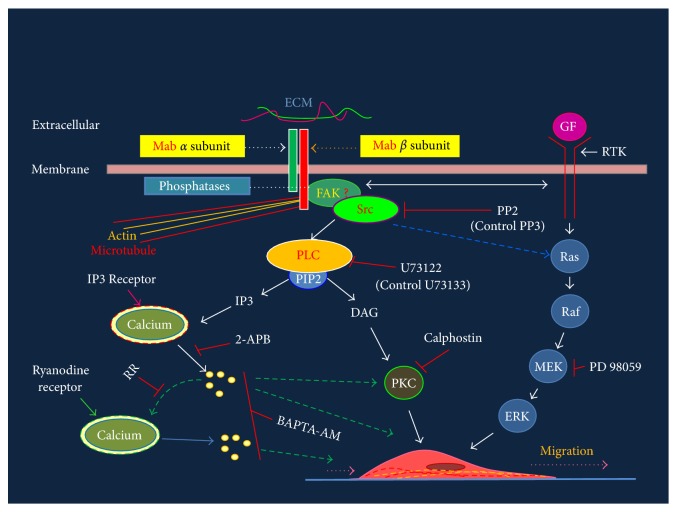
Schematic of integrin signaling cascade and its perturbation with antibodies and pharmacological agents. Integrin subunits (red and green bars on top) are shown intercalated in the membrane and interacting with ECM molecule (red and green horizontal lines). Molecules involved in signaling events are labeled and arrows point to the directions of signaling that starts with the engagement of integrin subunit with the extracellular matrix. Directions of calcium mediated signaling are shown by dashed green arrows. Inhibitors (see Table 2) used for blocking signaling molecules and paths are shown by red blocks. Cross-talk between integrin and GF signaling is shown by double headed horizontal arrow close to membrane. Mab: monoclonal antibody, GF: growth factor, RTK: receptor tyrosine kinase, MEK: MAP kinase kinase, PLC: phospholipase C, PIP2: phosphotidal inositol biphosphate, IP3: inositol (1,4,5)-triphosphate (IP3), DAG: diacylglycerol and PKC: protein kinase C, Ras: G protein, Raf: MAPKKK, MEK: MAPKK, ERK: extracellular signal regulated kinase (MAPK).

Rat fetal cortical neurons (isolated at GD16.5) are fully equipped with the integrin receptor and the classical intracellular signaling cascade (Figure 10) to maneuver their migration and neuritogenesis *in vitro*. Precisely, the laminin-binding *α*3*β*1 integrin receptor and intracellular signaling molecules drive both the migration and neuritogenesis while the cross-talk of integrin with growth factor signaling may add to more robust signaling towards the differentiation process. The detection of *α*9 subunit transcripts in fetal cortical neurons indicates that migration of fetal cortical neurons on radial glia may be supported by tenascin via the *α*9*β*1 integrin receptor. These results suggest that the composition of extracellular matrix in the path of migration and specific repertoire of integrin receptors regulates the dynamics of migration of neuronal precursors in the developing brains. The present study also indicates that calcium may regulate the migration of fetal cortical neurons also by maintaining the optimum supply of microtubules.

Additional studies are now required to delineate the exclusive role of each downstream molecule in the “outside-in” and “inside-out” integrin signaling that mediates the migration and neuritogenesis of fetal cortical neurons. This includes estimating the surface expression of each integrin subunit by flow cytometry and to examine the relevant concentrations of each antibody in the presence or absence of different combinations of pharmacological inhibitors as well as siRNA manipulation of components of integrin and growth factor signaling. Moreover, restoring the migration and neuritogenesis of neurons by activating different kinases following their inhibition by antibodies and/or pharmacological inhibitors may also be required to further ascertain their role. 

Additional studies are also required to identify the PKC isoform/s that regulates the migration and neuritogenesis of fetal cortical neurons, the roles of *α*1 and *α*9 integrin subunits, that are the constituent of laminin-binding *α*1*β*1 and tenascin-binding *α*9*β*1 integrin receptors respectively, the manipulation of molecules that regulate cross-talk between integrin and growth factor signaling (such as ILK, Paxillin, and PINCH), and the Semaphorins (specifically of Semaphorin 7A) that influence axon outgrowth and activation of MAPK [72]. 

Finally, in the perspective of results obtained by experimenting directly with fetal cortical neurons in culture and those describing no changes in the migration of neurons in *β*1 integrin mutant (*in vivo* studies) mice [18, 19], it is conceivable that the acute effects of monoclonal antibody on the neurons in culture (an *in vitro* test) may not be compensated which may occur in gene manipulation (*in vivo*) studies. It is also possible that the migration machinery of fetal cortical neurons in mice, which was used for gene manipulation studies by other investigators, may not be exactly similar to rat that was used in the current study. Even though the findings of *in vitro* studies presented here have their limitations, conclusions of previous gene manipulation studies [17–19] indicating that the *β*1 subunit is involved in glial differentiation but not in the glial-guided migration of neurons may not be stringent as data presented here show that fetal cortical neurons do utilize *β*1 and *α*3 integrin subunits and the intracellular molecules, that are known to support integrin signaling, for their migration. 

## Figures and Tables

**Table 1:** Antibodies, commercial sources, and applications.

Antibody	Source	Experiments
Monoclonal antibodies against *β*1 integrin subunit (Clone Ha2/5)	BD Biosciences (San Jose, CA)	Migration assays
Monoclonal antibodies against *β*3 integrin subunit (Clone 25E11)	Chemicon International (Temecula, CA)	Migration assays
Monoclonal antibodies against *α*3 integrin subunit (Clone P1B5)	Chemicon International	Migration assays
Monoclonal antibodies against *α*4 integrin subunit (Clone R1-2)	BD Biosciences	Migration assays
Monoclonal antibodies against *α*5 integrin subunit (Clone VC5)	BD Biosciences	Migration assays
Monoclonal antibodies against *αv* integrin subunit (Clone H9.2B8)	BD Biosciences	Migration assays
Monoclonal antibodies against *α*6 integrin subunit (Clone NKI-GoH3)	BD Biosciences	Migration assays
Control IgG (Clone A19-4)	BD Biosciences	Migration assays
Control IgM (Clone G235-1)	BD Biosciences	Migration assays
Monoclonal antibody against NeuN (Clone A60)	Chemicon International	Immunofluorescent microscopy
Rabbit Polyclonal antibody against MAP2 (AB5622)	Chemicon International	Immunofluorescent microscopy
Monoclonal Antibody against microtubules (E7)	Hybridoma Bank (University of Iowa, IA)	Immunofluorescent microscopy
Monoclonal antibody against *β*-Actin (Clone C4)	BD Biosciences	Western blotting
Purified mouse anti (pTyr783)-Phospolipase C *γ*1 Clone 27/Phospholipase C-*γ* (pY783)	BD Biosciences	Western blotting
Alexa Fluor 594-conjugated secondary antibodies (Donkey anti-mouse or rabbit)	Invitrogen, Carlsbad, CA	Immunofluorescence microscopy
Peroxidase-conjugated secondary antibodies (Donkey anti-mouse)	Jackson Immuno-research, West Grove, PA	Western blotting

**Table 2:** Inhibitors, intracellular calcium regulators and targets.

Inhibitor	Target	Concentration/references
4-Amino-5-(4-chlorophenyl)-7-(t-butyl) parasol [3,4-] pyrimidine (PP2) and control compound (PP3)	Src family of tyrosine kinases	20 *μ*M [32]
1-[6-((17*β*-3-Methoxyestra-1,3,5(10)-trien-17-y) amino) hexyl]-1H-pyrrole-2,5-dione (U-73122 or U2) and control compound U-73343 or U3	Phospholipase C activation	10 *μ*M [33]
2′Amino-3′-Methoxyflavone (PD98059 or PD)	MAP kinase kinase	20 *μ*M [34]
2-(Aminoethoxy) diphenylborate (2-APB)	Ins (1,4,5)P_3_-induced Ca^2^ release	40 and 100 *μ*M [35]
1,2-bis(o-Aminophenoxy) ethane-N,N,N′,N′-tetraacetic acid tetra (BAPTA/AM)	Calcium chelator	2.5 and 10 *μ*M [36]
Calphostin C (UCN-1028c)	Protein kinase C	50 nM [37]
Ammoniated Ruthenium Oxychloride (RR)	Calcium-induced calcium release from Ryanodine-sensitive intracellular calcium stores	50 *μ*M [38]

**Table 3:** Primers used for the amplification of integrin cDNA from rat fetal brain neurons.

Subunit	Sense primer (5′-3′)	Antisense primer (5′-3′)	Amplicon size
*β*1	aatgtttcagtgcagagcc	ttgggatgatgtcgggac	261
*β*3	agctgtcgctgtccttcaat	cctgctgagagggtcgatag	357
*β*4	gctctacacggacaccacct	tgcagcaggcacagtatttc	398
*α*1	tggatattggccctaagcag	cgcttgcgatcgattttatt	399
*α*2	tggggtgcaaacagacaagg	gtaggtctgctggttcagc	539
*α*3	ctgctgccaaaaaagccaagt	ggcagctcctccaccagct	300
*α*4	cccaggctacatcgttttgt	atggtcttcatgctcccaac	444
*α*5	aggtgacgggactcaacaac	agccgagcttgtagaggaca	489
*αv *	gagcagcaaggactttggg	gggtacacttcaaggccagc	619
*α*6	gactcttaactgtagcgtga	atctctcgctcttctttccg	550 (*α*6A), 420 (*α*6B)
*α*9	tcccccagtactcgatgaag	cagtctctcccagcaacaca	396

## References

[1] Métin C., Baudoin J. P., Rakić S., Parnavelas J. G. (2006). Cell and molecular mechanisms involved in the migration of cortical interneurons. *European The Journal of Neuroscience*.

[2] Gupton S. L., Gertler F. B. (2010). Integrin signaling switches the cytoskeletal and exocytic machinery that drives neuritogenesis. *Developmental Cell*.

[3] Anton E. S., Kreidberg J. A., Rakic P. (1999). Distinct functions of *α*3 and *α*(v) integrin receptors in neuronal migration and laminar organization of the cerebral cortex. *Neuron*.

[4] Campbell I. D., Humphries M. J. (2011). Integrin structure, activation, and interactions. *Cold Spring Harbor Perspectives in Biology*.

[5] Qin J., Vinogradova O., Plow E. F. (2004). Integrin bidirectional signaling: a molecular view. *PLoS Biology*.

[6] Milner R., Campbell I. L. (2002). The integrin family of cell adhesion molecules has multiple functions within the CNS. *Journal of Neuroscience Research*.

[7] Barczyk M., Carracedo S., Gullberg D. (2010). Integrins. *Cell and Tissue Research*.

[8] Reichardt L. F., Tomaselli K. J. (1991). Extracellular matrix molecules and their receptors: functions in neural development. *Annual Review of Neuroscience*.

[9] Schmid R. S., Anton E. S. (2003). Role of integrins in the development of the cerebral cortex. *Cerebral Cortex*.

[10] Schmid R. S., Shelton S., Stanco A., Yokota Y., Kreidberg J. A., Anton E. S. (2004). *α*3*β*1 integrin modulates neuronal migration and placement during early stages of cerebral cortical development. *Development*.

[11] Georges-Labouesse E., Mark M., Messaddeq N., Gansmüller A. (1998). Essential role of *α*6 integrins in cortical and retinal lamination. *Current Biology*.

[12] Marchetti G., Escuin S., van der Flier A., de Arcangelis A., Hynes R. O., Georges-Labouesse E. (2010). Integrin *α*5*β*1 is necessary for regulation of radial migration of cortical neurons during mouse brain development. *European The Journal of Neuroscience*.

[13] Galileo D. S., Majors J., Horwitz A. F., Sanes J. R. (1992). Retrovirally introduced antisense integrin RNA inhibits neuroblast migration in vivo. *Neuron*.

[14] Andressen C., Adrian S., Fässler R., Arnhold S., Addicks K. (2005). The contribution of *β*1 integrins to neuronal migration and differentiation depends on extracellular matrix molecules. *European Journal of Cell Biology*.

[15] DeFreitas M. F., Yoshida C. K., Frazier W. A., Mendrick D. L., Kypta R. M., Reichardt L. F. (1995). Identification of integrin *α*3*β*1 as a neuronal thrombospondin receptor mediating neurite outgrowth. *Neuron*.

[16] Muller U., Bossy B., Venstrom K., Reichardt L. F. (1995). Integrin *α*8*β*1 promotes attachment, cell spreading, and neurite outgrowth on fibronectin. *Molecular Biology of the Cell*.

[17] Belvindrah R., Graus-Porta D., Goebbels S., Nave K. A., Müller U. (2007). *β*1 integrins in radial glia but not in migrating neurons are essential for the formation of cell layers in the cerebral cortex. *The Journal of Neuroscience*.

[18] Fassler R., Meyer M. (1995). Consequences of lack of *β*1 integrin gene expression in mice. *Genes and Development*.

[19] Graus-Porta D., Blaess S., Senften M. (2001). *β*1-class integrins regulate the development of laminae and folia in the cerebral and cerebellar cortex. *Neuron*.

[20] Förster E., Tielsch A., Saum B. (2002). Reelin, disabled 1, and *β*1 integrins are required for the formation of the radial glial scaffold in the hippocampus. *Proceedings of the National Academy of Sciences of the United States of America*.

[21] Niewmierzycka A., Mills J., St.-Arnaud R. R., Dedhar S., Reichardt L. F. (2005). Integrin-linked kinase deletion from mouse cortex results in cortical lamination defects resembling cobblestone lissencephaly. *The Journal of Neuroscience*.

[22] Belvindrah R., Nalbant P., Ding S., Wu C., Bokoch G. M., Müller U. (2006). Integrin-linked kinase regulates Bergmann glial differentiation during cerebellar development. *Molecular and Cellular Neuroscience*.

[23] Nikonenko I., Toni N., Moosmayer M., Shigeri Y., Muller D., Jones L. S. (2003). Integrins are involved in synaptogenesis, cell spreading, and adhesion in the postnatal brain. *Developmental Brain Research*.

[24] Liesi P., Seppala I., Trenkner E. (1992). Neuronal migration in cerebellar microcultures is inhibited by antibodies against a neurite outgrowth domain of laminin. *Journal of Neuroscience Research*.

[25] Zassler B., Schermer C., Humpel C. (2003). Protein kinase C and phosphoinositol-3-kinase mediate differentiation or proliferation of slice-derived rat microglia. *Pharmacology*.

[26] Schermer C., Humpel C. (2002). Granulocyte macrophage-colony stimulating factor activates microglia in rat cortex organotypic brain slices. *Neuroscience Letters*.

[27] Stettler E. M., Galileo D. S. (2004). Radial glia produce and align the ligand fibronectin during neuronal migration in the developing chick brain. *Journal of Comparative Neurology*.

[28] Liesi P., Dahl D., Vaheri A. (1983). Laminin is produced by early rat astrocytes in primary culture. *Journal of Cell Biology*.

[29] Aplin A. E., Howe A., Alahari S. K., Juliano R. L. (1998). Signal transduction and signal modulation by cell adhesion receptors: the role of integrins, cadherins, immunoglobulin-cell adhesion molecules, and selectins. *Pharmacological Reviews*.

[30] Juliano R. L., Reddig P., Alahari S., Edin M., Howe A., Aplin A. (2004). Integrin regulation of cell signalling and motility. *Biochemical Society Transactions*.

[31] Eliceiri B. P. (2001). Integrin and growth factor receptor crosstalk. *Circulation Research*.

[32] Hsia D. A., Lim S. T., Bernard-Trifilo J. A. (2005). Integrin *α*4*β*1 promotes focal adhesion kinase-independent cell motility via *α*4 cytoplasmic domain-specific activation of c-Src. *Molecular and Cellular Biology*.

[33] Bleasdale J. E., Thakur N. R., Gremban R. S. (1990). Selective inhibition of receptor-coupled phospholipase C-dependent processes in human platelets and polymorphonuclear neutrophils. *Journal of Pharmacology and Experimental Therapeutics*.

[34] Gleeson L. M., Chakraborty C., Mckinnon T., Lala P. K. (2001). Insulin-like growth factor-binding protein 1 stimulates human trophoblast migration by signaling through *α*5*β*1 integrin via mitogen-activated protein kinase pathway. *Journal of Clinical Endocrinology and Metabolism*.

[35] Ma H. T., Venkatachalam K., Li H. S. (2001). Assessment of the role of the inositol 1,4,5-trisphosphate receptor in the activation of transient receptor potential channels and store-operated Ca^2+^ entry channels. *Journal of Biological Chemistry*.

[36] Hirai K., Yoshioka H., Kihara M. (1999). Inhibiting neuronal migration by blocking NMDA receptors in the embryonic rat cerebral cortex: a tissue culture study. *Developmental Brain Research*.

[37] Ranta-Knuuttila T., Kiviluoto T., Mustonen H. (2002). Migration of primary cultured rabbit gastric epithelial cells requires intact protein kinase C and Ca^2+^/calmodulin activity. *Digestive Diseases and Sciences*.

[38] Phillippe M., Basa A. (1996). The effects of ruthenium red, an inhibitor of calcium-induced calcium release, on phasic myometrial contractions. *Biochemical and Biophysical Research Communications*.

[39] Maeda N., Noda M. (1998). Involvement of receptor-like protein tyrosine phosphatase *ζ*/RPTP*β* and its ligand pleiotrophin/heparin-binding growth-associated molecule (HB-GAM) in neuronal migration. *Journal of Cell Biology*.

[40] Rout U. K., Dhossche J. M. (2010). Liquid-diet with alcohol alters maternal, fetal and placental weights and the expression of molecules involved in integrin signaling in the fetal cerebral cortex. *International Journal of Environmental Research and Public Health*.

[41] Aarum J., Sandberg K., Haeberlein S. L. B., Persson M. A. A. (2003). Migration and differentiation of neural precursor cells can be directed by microglia. *Proceedings of the National Academy of Sciences of the United States of America*.

[42] Segarra J., Balenci L., Drenth T., Maina F., Lamballe F. (2006). Combined signaling through ERK, PI3K/AKT, and RAC1/p38 is required for met-triggered cortical neuron migration. *Journal of Biological Chemistry*.

[43] Borghesani P. R., Peyrin J. M., Klein R. (2002). BDNF stimulates migration of cerebellar granule cells. *Development*.

[44] Mendrick D. L., Kelly D. M. (1993). Temporal expression of VLA-2 and modulation of its ligand specificity by rat glomerular epithelial cells in vitro. *Laboratory Investigation*.

[45] Ferguson T. A., Kupper T. S. (1993). Antigen-independent processes in antigen-specific immunity: a role for *α*4 integrin. *Journal of Immunology*.

[46] van Nhieu G. T., Isberg R. R. (1991). The Yersinia pseudotuberculosis invasin protein and human fibronectin bind to mutually exclusive sites on the *α*5*β*1 integrin receptor. *Journal of Biological Chemistry*.

[47] Moulder K., Roberts K., Shevach E. M., Coligan J. E. (1991). The mouse vitronectin receptor is a T cell activation antigen. *Journal of Experimental Medicine*.

[48] Aumailley M., Timpl R., Sonnenberg A. (1990). Antibody to integrin *α*6 subunit specifically inhibits cell-binding to laminin fragment 8. *Experimental Cell Research*.

[49] Burns G. F., Cosgrove L., Triglia T. (1986). The IIb-IIIa glycoprotein complex that mediates platelet aggregation is directly implicated in leukocyte adhesion. *Cell*.

[50] Wayner E. A., Carter W. G., Piotrowicz R. S., Kunicki T. J. (1988). The function of multiple extracellular matrix receptors in mediating cell adhesion to extracellular matrix: preparation of monoclonal antibodies to the fibronectin receptor that specifically inhibit cell adhesion to fibronectin and react with platelet glycoproteins Ic-IIa. *Journal of Cell Biology*.

[51] Rout U. K. (2006). Valproate, thalidomide and ethyl alcohol alter the migration of HTR-8/SVneo cells. *Reproductive Biology and Endocrinology*.

[52] Maitra N., Flink I. L., Bahl J. J., Morkin E. (2000). Expression of *α* and *β* integrins during terminal differentiation of cardiomyocytes. *Cardiovascular Research*.

[53] Feltri M. L., Arona M., Scherer S. S., Wrabetz L. (1997). Cloning and sequence of the cDNA encoding the *β*4 integrin subunit in rat peripheral nerve. *Gene*.

[54] Condic M. L., Letourneau P. C. (1997). Ligand-induced changes in integrin expression regulate neuronal adhesion and neurite outgrowth. *Nature*.

[55] Palecek S. P., Loftust J. C., Ginsberg M. H., Lauffenburger D. A., Horwitz A. F. (1997). Integrin-ligand binding properties govern cell migration speed through cell-substratum adhesiveness. *Nature*.

[56] Tucker R. P., Brunso-Bechtold J. K., Jenrath D. A. (1994). Cellular origins of tenascin in the developing nervous system. *Perspectives on Developmental Neurobiology*.

[57] Seiffert D., Iruela-Arispe M. L., Sage E. H., Loskutoff D. J. (1995). Distribution of vitronectin mRNA during murine development. *Developmental Dynamics*.

[58] McCarty J. H., Lacy-Hulbert A., Charest A. (2005). Selective ablation of *α*v integrins in the central nervous system leads to cerebral hemorrhage, seizures, axonal degeneration and premature death. *Development*.

[59] Schottelndreier H., Potter B. V., Mayr G. W., Guse A. H. (2001). Mechanisms involved in alpha6beta1-integrin-mediated Ca(2+) signalling. *Cellular Signalling*.

[60] Fukami K., Inanobe S., Kanemaru K., Nakamura Y. (2010). Phospholipase C is a key enzyme regulating intracellular calcium and modulating the phosphoinositide balance. *Progress in Lipid Research*.

[61] Choi J. H., Yang Y. R., Lee S. K. (2007). Phospholipase C-*γ*1 potentiates integrin-dependent cell spreading and migration through Pyk2/paxillin activation. *Cellular Signalling*.

[62] Larsson C. (2006). Protein kinase C and the regulation of the actin cytoskeleton. *Cellular Signalling*.

[63] Zheng J. Q., Poo M. M. (2007). Calcium signaling in neuronal motility. *Annual Review of Cell and Developmental Biology*.

[64] Zhang X., Chattopadhyay A., Ji Q. S. (1999). Focal adhesion kinase promotes phospholipase C-*γ*1 activity. *Proceedings of the National Academy of Sciences of the United States of America*.

[65] Jones N. P., Peak J., Brader S., Eccles S. A., Katan M. (2005). PLC*γ*1 is essential for early events in integrin signalling required for cell motility. *Journal of Cell Science*.

[66] Zhou L., Jossin Y., Goffinet A. M. (2007). Identification of small molecules that interfere with radial neuronal migration and early cortical plate development. *Cerebral Cortex*.

[67] Zhao C. T., Li K., Li J. T. (2009). PKC*δ* regulates cortical radial migration by stabilizing the Cdk5 activator p35. *Proceedings of the National Academy of Sciences of the United States of America*.

[68] Ng T., Shima D., Squire A. (1999). PKC*α* regulates *β*1 integrin-dependent cell motility through association and control of integrin traffic. *The EMBO Journal*.

[69] Alam N., Goel H. L., Zarif M. J. (2007). The integrin—growth factor receptor duet. *Journal of Cellular Physiology*.

[70] Umesh A., Thompson M. A., Chini E. N., Yip K. P., Sham J. S. K. (2006). Integrin ligands mobilize Ca^2+^ from ryanodine receptor-gated stores and lysosome-related acidic organelles in pulmonary arterial smooth muscle cells. *Journal of Biological Chemistry*.

[71] O'Brien E. T., Salmon E. D., Erickson H. P. (1997). How calcium causes microtubule depolymerization. *Cell Motility and the Cytoskeleton*.

[72] Pasterkamp R. J., Peschon J. J., Spriggs M. K., Kolodkin A. L. (2003). Semaphorin 7A promotes axon outgrowth through integrins and MAPKs. *Nature*.

